# Predictors of work ability of secondary school teachers in Germany

**DOI:** 10.3389/fpubh.2025.1708490

**Published:** 2026-02-11

**Authors:** Steffi Kreuzfeld, Reingard Seibt

**Affiliations:** Institute for Occupational, Social, and Environmental Medicine, Rostock University Medical Center, University of Rostock, Rostock, Germany

**Keywords:** age group, effort-reward imbalance, emotional exhaustion, overcommitment, predictor, secondary school teacher, work ability, work ability index factor 1

## Abstract

**Objectives:**

Maintaining work ability is essential for teachers to remain in the profession for longer periods. This study examines whether work ability can be predicted by work-related and personal factors.

**Methods:**

The data comes from a representative cross-sectional study of German secondary school teachers, which included 10,739 full-time and part-time teachers (68% female, average age: 43 years). Work ability was measured using the Work Ability Index (WAI) and WAI factor 1. The teachers were divided into three age groups: 31–40 (*n*=4,514), 41–50 (*n*=3,925) and 51–60 years (*n*=2,300). The predictability of work ability was analyzed using regression models for the following factors: effort-reward ratio (ER ratio), overcommitment (OC), emotional exhaustion (EE), and age. CHAID analyses were also performed to investigate the complex associations between these variables.

**Results:**

In the overall models, the four factors explain 42% (WAI) and 46% (WAI factor 1) of the variance. EE proved to be the most important predictor of work ability, explaining 36% (WAI) and 42% (WAI factor 1) of the variance; ER ratio and OC explained only 15% to 18% of the variance. Age was a significant predictor, but of minor importance. In the CHAID decision tree, teachers with high EE and an ER imbalance (ERI) in the 51–60 age group showed the lowest work ability (WAI: M=33 out of 49 pts, 10%). The best work ability (*M*=42 out of 49 pts) was found among younger teachers (31–40 years) with a normal ER ratio and normal EE and OC values (18%). For WAI factor 1, the worst mean value (*M*=19 out of 31 pts) was seen among teachers with high EE and ERI in the 41–60 age group, while the best work ability (*M*=25 out of 31 pts) was observed among teachers with normal values for EE, ER ratio, and OC in the age group from 31 to 50 years (33% of the sample).

**Conclusion:**

In occupational health prevention programs, WAI factor 1, EE, and ER ratio are suitable tools for advising teachers on how to maintain their work ability.

## 1 Introduction

Teachers have a demanding job that is characterized by a variety of cognitive, social, and emotional requirements and needs strong professional time management skills (1, 2). In addition to their workload, many teachers complain about time pressure, inadequate support from colleagues and school management, conflicts with pupils and parents, and a lack of recognition and appreciation (3, 4). Compared to other employees, teachers report emotional and physical exhaustion more frequently (5, 6). A significant proportion of German teachers (42%) voluntarily reduce their teaching hours in order to cope with the demands of their job (7). Nevertheless, the proportion of teachers who retire early is high compared to other occupational groups (8). The main reasons cited are excessive stress, but also mental illness, frustration, and insufficient rest (9, 10).

To help teachers stay healthy in their profession for as long as possible, the concept of work ability (11) has become established in research. It describes the ability of individuals to meet the physical and psychosocial demands of work by using personal and organizational resources. This interplay between demands and resources is dynamically influenced by numerous factors, such as physical and mental health, skills, motivation, working conditions, and the work environment. Various studies have also observed that work ability declines with age (12–15) and that there are associations with psychosocial and organizational work factors (12, 16–19). For example, high effort and destructive leadership behavior have a negative impact on work ability (19). In the long term, lower perceived work ability has been associated with an increased risk of long-term sickness absence and early retirement (16, 20–22).

The effort-reward imbalance (ERI) model (23) has become particularly important in assessing the health effects of working conditions and psychosocial workload. An imbalance between the effort required at work and the material and non-material rewards received has been identified as a psychosocial stress factor that increases the risk of reduced work ability (24) and stress-related health problems (25, 26). Former studies have shown that teachers experience a greater imbalance between effort and reward and greater exhaustion than other occupational groups (5, 27, 28).

The ERI model also includes the intrinsic component of overcommitment, which describes a person’s tendency to accomplish work tasks through excessive commitment, even without adequate reward and at the expense of potential health risks (23). Employees with this coping pattern have difficulty detaching themselves from their work. They are therefore more susceptible to exhaustion and burnout (29, 30). Longitudinal studies have shown that overcommitment (OC) in combination with other stressors and poor health is a predictor of reduced work ability among healthcare workers (31, 32).

Like the ERI model, teachers’ burnout syndrome has become particularly important in explaining their work ability (33, 34). The syndrome is a reaction to chronic occupational stress. In addition, individual characteristics and work behavior are considered to be causes of burnout (35). According to Maslach et al. (36), it comprises three subscales: ‘emotional exhaustion’, ‘depersonalization’ or ‘cynicism’, and ‘personal accomplishment’. Emotional exhaustion (EE) is considered the main component of burnout syndrome and is one of the most common risks that affect teachers’ well-being (37, 38).

Although the burnout syndrome has been studied frequently in teachers (39, 40), it has rarely been investigated in association with work ability. The earlier study by Seibt et al. (5) associated good work ability among female secondary school teachers with a low risk of burnout. Associations between work ability and burnout or emotional exhaustion have also been found among university employees (33). Hlado et al. (34) identified burnout as an important predictor of work ability with a strong negative effect among Czech secondary school teachers. Otherwise, Paudel et al. (41) emphasized work ability as a significant factor that is related to all three subscales of burnout among teachers. In summary, a review of previous research on work ability highlighted the interaction of work ability as both a predictor and a result of various variables (e.g., job satisfaction, mental health) (42).

The Work Ability Index (WAI) developed by Tuomi et al. (43) is considered a globally used tool with high practical relevance, as it provides entry points for prevention and intervention in the workplace. Schouteten et al. (33) assessed the WAI as a possible tool for identifying individuals at risk of occupational burnout.

However, the originally accepted one-dimensionality of the unweighted WAI (sum score) has not been verified in different studies. Research on the validity of the WAI indicated that its predictive power was generated in particular from items that were not explicitly related to health (44). Currently, a two-factor structure is assumed (42, 45–47). This was confirmed in a representative study by Freyer et al. (48), according to which the indicators WAI 1, WAI 2, WAI 6, and WAI 7 represent the WAI factor 1 “current and future work ability and individual resources”. The second factor, on the other hand, represents an individual health related factor with the indicators WAI 3, WAI 4, and WAI 5 (48). The representative sample also yielded age-related reference values for both factors for the work ability of the German working population.

To our knowledge, no systematic analyses of teachers’ work ability based on the two-factor structure of the WAI have been conducted to date, nor have possible associations with work-related and personal predictors been investigated. Earlier studies on the work ability of secondary school teachers (TE) showed that the majority of them (75% of those under 45 and 57% of those over 45 years) rated their own work ability as good or excellent (5). However, compared to office workers (OW), more than twice as many teachers (37% vs. 18%) reported poor or moderate work ability, while the opposite was true for excellent work ability (TE vs. OW: 13% vs. 28%). Good work ability was almost equally distributed in both occupational groups (50% vs. 53%) (5).

The study raises the following questions:

1. What is the age-related work ability (WAI, WAI Factor 1) of teachers compared to the work ability of the reference sample from the German working population?
2. Is there an association between work-related (ERI) and personal factors (OC, EE) and the work ability of teachers?
3. Are these work-related and personal factors suitable predictors of work ability?

## 2 Methods

### 2.1 Implementation and recruitment

The data were collected between January and April 2018 as part of the nationwide cross-sectional study ‘Lehrerarbeit im Wandel’ (LaiW study). The study examined the workload, work ability, and health of teachers at secondary schools in Germany. A detailed description of the study design, sampling, and implementation can be found in Kreuzfeld et al. (10). The LaiW study meets the requirements for representativeness in terms of gender, age, and teaching workload of secondary school teachers (hereinafter referred to as teachers) in Germany.

In the run-up to the study, flyers were distributed at all secondary schools to encourage voluntary participation. Before the study began, all teachers received information on data protection, the implementation and evaluation of the study, the conditions of participation, and access to the study. The anonymity of the data was ensured by transaction numbers and an eight-digit personal code. Data was collected via an online portal of the University of Rostock.

The design and all details of the study were approved by the Local Ethics Committee (A 2018–0031). Informed consent was given by every participant prior to inclusion in the study.

### 2.2 Measures

The study consisted of an online questionnaire (OF) and an online protocol (OP). Both procedures were developed and applied at the Rostock University Medical Center. The data from both the OF and the OP were merged using the personal code. Only data from participants for whom an OF and an OP were available were included in the data analysis.

#### 2.2.1 Online questionnaire

The online questionnaire consisted of standardized questionnaires on work ability, psychosocial workload, overcommitment, and emotional exhaustion, as well as self-developed questions on sociodemographic (e.g., gender, age, marital status) and job-specific information (e.g., teaching obligations, subjects taught, classes, number of students).

##### 2.2.1.1 Work ability

Work ability was assessed using the short version of the German Work Ability Index (WAI) questionnaire, which consists of 10 questions (each with one to three items) and 14 disease categories. These are summarized in the following seven WAI subscales (WAI 1 - WAI 7): WAI 1 - current work ability compared with the lifetime best, WAI 2 - work ability in relation to the demands of the job, WAI 3 - current diseases diagnosed by a physician, WAI 4 - estimated work impairment due to diseases, WAI 5 - sick leave during the past year (12 months), WAI 6 - own prognosis of work ability 2 years from now, WAI 7 - mental resources (49).

The items in the WAI are heterogeneous and have different response formats. The WAI was evaluated according to the guidelines in the manual (49), whereby the points from the seven subscales are summarized unweighted (exception: WAI 2) to form the WAI. The WAI can take values between 7 and 49 points (pts) and can be classified as poor (7–27 pts), moderate (28–36 pts), good (37–43 pts), and excellent (44–49 pts) work ability.

Based on the two-factor structure of Freyer et al. (47), *‘*work ability and resources’ are mapped in the WAI factor 1 subscale (WAI F1), which is derived from the subscales WAI 1, WAI 2, WAI 6, and WAI 7 and represents the weighted sum of these subscales in poor (4–22 pts), moderate (23–26 pts), good (27–30 pts) and excellent (31 pts) work ability. The scoring is based on the assumption by Tuomi et al. (50) that 15% of a sample fall into the poor and excellent categories and 30% into the good and moderate categories.

The individual test score can be classified using a norm-referenced scores Table (31–40, 41–50, 51–60 years) that represents the German working population (48). The reference sample comes from the ‘Study on Mental Health at Work’, which was initiated by the Federal Institute for Occupational Safety and Health and conducted in cooperation with the Institute for Employment Research of the Federal Employment Agency and the Institute for Applied Social Sciences GmbH (51).

For the WAI of German samples, Cronbach’s alpha values ranging from *α* = 0.58–0.77 (46) and *α* = 0.83 (52) were reported, which, according to Blanz (53), vary between poor and good internal consistency. Freyer et al. (47) reported a Cronbach’s alpha of 0.70 for the reliability of the WAI factor 1. For the WAI and the WAI factor 1 in the present study, Cronbach’s alpha was 0.77 and 0.75, which is within the acceptable range according to Blanz (53).

##### 2.2.1.2 Psychosocial workload

Psychosocial workload was assessed using the short form of the Effort-Reward Imbalance Questionnaire (ERI-Q) (23). This questionnaire enables a standardized measurement of the imbalance between work-related effort and reward (effort-reward ratio (ER ratio)) and comprises the main scales effort (three items; range: 3–15 pts) and reward (seven items; range: 7–35 pts). The reward scale consists of the three subscales status (salary, job promotion), esteem and job security. High totals on the main scales indicate high perceived effort or reward.

The ER ratio is calculated from the sum of the values of the two main subscales (effort and reward) using the following formula: ER ratio = ∑ effort / (∑ reward * 0.54). An ER ratio of > 1 indicates an effort-reward imbalance (ERI) (23), which is associated with a health risk. The greater the imbalance between effort and reward, the higher the health risk is said to be.

Validity and reliability are acceptable for the main scales of the short version of the ERI-Q (23, 53); Cronbach’s alphas above 0.70 were reported (effort: 0.74, reward: 0.79). For the reward subscale, a Cronbach’s alpha of 0.71 was also determined in the present study, which is considered acceptable internal consistency (53). For the effort scale, however, Cronbach’s alpha was only 0.52, indicating low internal consistency (53).

##### 2.2.1.3 Overcommitment

Overcommitment (OC) is also part of the ERI-Q (23) and represents the intrinsic component. It comprises six items that are rated on a four-point Likert scale (1 = strongly disagree to 4 = strongly agree). The six items are summed up to give a total score (range: 6–24 pts), with high OC scores indicating a high tendency to overexert oneself. The upper tertile of the OC score is defined as the risk group (23).

Siegrist et al. (23) reported a Cronbach’s alpha of 0.79 for all subscales of the OC in the short version of the ERI-Q, which corresponds to acceptable internal consistency (53). In the present study, a Cronbach’s alpha of 0.77 was calculated for OC, which is also considered acceptable internal consistency (53).

##### 2.2.1.4 Emotional exhaustion

Emotional exhaustion (EE) as a core component of the Maslach Burnout Inventory-General Survey (MBI-GS) (54) was assessed using the German translation of the MBI-GS-D (55). This subscale measures the frequency of symptoms using five items on a seven-point Likert scale (0 = never to 6 = daily). The mean value is calculated from these five items and can be assigned to low (≤2.0 pts), normal (>2.0–3.2 pts) or high (>3.2–6.0 pts) emotional exhaustion (54). In this study, low and normal EE values were combined as normal EE.

The validity of the MBI-GS-D was demonstrated by Schaufeli et al. (55): Cronbach’s alpha for EE was 0.87, indicating high internal consistency (53). In the study presented here, Cronbach’s for EE was 0.85, thus demonstrating comparably high internal consistency (53).

#### 2.2.2 Online protocol

The online protocol was used to determine the average weekly working time of teachers. To this end, working time had to be documented daily over a period of 4 weeks (28 days) using 12 practical, teacher-specific activity categories (10). To calculate the weekly working time, the average value for each activity category was first determined over a period of 4 weeks. These average values were then added together. In the case of sick days, the average value for the weekly working time was determined from the remaining weeks; at least 21 days of working time had to be documented.

### 2.3 Data control and statistical analyses

Prior to the statistical calculations, the entire data set was checked for implausible entries. Data sets with missing values (6%) were excluded in advance. With regard to the average working time per week, extreme values within a single activity category were replaced by subject-specific averages. The number of teaching hours and reduced teaching hours was checked in the OQ based on information on the teachers’ age and special tasks.

The statistical analysis of the data was performed using the Statistical Package for the Social Sciences (SPSS, version 28).

Mean differences between age groups were examined for metric factors (variables) using univariate covariance analyses. Only gender was included as a control variable in these variance analyses. The *χ*^2^-test was used for categorical variables. The significance of the working ability between the sample of teachers and the reference sample (48) was tested using the one sample *t*-test.

A probability of error of 5% was set as the statistical significance criterion and supplemented by effect sizes (56). The interpretation of the effect sizes was based on the conventions of Cohen (57). Small effect sizes are considered to *η*^2^_p_ ≥ 0.01 (covariance analyses) and *d* ≥ 0.20 (*χ*^2^-tests).

Correlations between work ability and work-related and personal factors or age were examined using Spearman’s rank correlation coefficient (*R*) and interpreted according to Bühl (58). To examine these correlations with gender, the Eta coefficient (*η*) was calculated and interpreted according to Cohen (57). Correlation coefficients ≤± 0.10 were considered independent of each other.

To clarify the influence of work-related and personal factors, including the control variable (independent variables – regressors), on work ability (dependent variable – criterion), simple and multiple linear regression analyses were calculated, with all independent variables included in the model simultaneously (method: enter). The selection of predictors included in the overall model was based on the results of the correlation analysis and simple linear regression. The *F*-test (ANOVA) was used to check whether the regression model was significant overall. The corrected coefficient of determination *R*^2^ was implemented to assess the goodness-of-fit of the model.

In addition, CHAID analyses (chi-squared automatic interaction detector analyses) (59) were performed. The CHAID analysis is a decision-tree algorithm based on chi-square statistics and preferably integrates categorical data. It shows how the various predictors affect the target criterion (WAI, WAI F1). The algorithm generates splits at a so-called node so that a variable can be divided into at least two categories. This results in decision trees that clearly illustrate the complex relationships between the target criterion and the predictors. The algorithm uses Bonferroni corrections. As a result, the top node of a CHAID tree represents the entire sample. In the levels below, the total sample is divided into more homogeneous groups that differ significantly in terms of the mean values of the criterion (WAI, WAI F1). The predictors (explanatory variables: χ^2^-tests) are used to distinguish between these groups.

### 2.4 Sample

A total of more than 20,000 secondary school teachers took part in the LaiW study. Of these, 18,791 completed the entire OQ. The quality requirements in the OQ and OP were met by 14,338 participants. The data for the present analysis was generated from this sample. To enable comparison with the reference sample of the Federal Institute for Occupational Safety and Health (47), only teachers aged between 31 and 60 were included in the study and assigned to the following three age groups (AG): AG_31-40_ (31–40 years, *n* = 4,514), AG_41-50_ (41–50 years, *n* = 3,925), AG_51-60_ (51–60 years, *n* = 2,300). This resulted in a sample size of 10,739 full-time and part-time teachers, whereby full-time in the teaching profession corresponds to the contractual teaching obligation and part-time is defined as any employment in which the regular teaching obligation is not met.

The sample consisted of approximately one-third men (32%) and two-thirds women (68%). Less than half of the teachers (42%) were under 40 years of age, and 58% were over 40. Approximately half of the teachers worked full-time (49%), with the highest proportion of full-time teachers in the AG_31-40_ (58%) and the lowest in the AG_41-50_ (36%) (*d* = 0.35 – small effect). The average age of teachers was 43 years (*SD* = 8 years) and was 35, 45, and 55 years in the three age groups (*SD* = 3 years).

The composition of the sample is shown in Table 1:

**Table 1:** Characteristics of sample of teachers.

Socio-demographic characteristic	Total sample		Age group [years]						Significance		
			31–40		41–50		51–60				
	*n*	%	*n*	%	*n*	%	*n*	%	Test value	*p*-value	Effect size
Number (%)	10,739	100	4,514	42.0	3,925	36.5	2,300	21.4			
Gender											
Male	3,432	32.0	1,540	44.9	1,229	35.8	663	19.3	20.80	<0.001	0.09
Female	7,307	68.0	2,974	40.7	2,696	36.9	1,637	22.4			
Extent of employment											
Full-time teachers	5,221	48.6	2,609	57.8	1,513	38.5	1,099	47.8	312.25	<0.001	0.35
Part-time teachers	5,518	51.4	1,905	42.2	2,412	61.5	1,201	52.2			
Family obligations											
Permanent partnership	9,146	85.2	3,757	83,2	3,424	87.2	1,965	85.4	26.83	<0.001	0.10
Children in the household	5,710	53.3	1,873	51.5	2,832	72.2	1,014	44.1	262.99	<0.001	0.32
Care of relatives	639	6.0	105	2.3	222	5.7	312	13.6	344.88	<0.001	0.36
Working time	*M ± SD*		*M ± SD*		*M ± SD*		*M ± SD*				
All teachers [hr/week]	40.6 ± 10.1		41.3 ± 10.6		39.3 ± 10.0		41.3 ± 9.3		49.37	<0.001	0.14
Full-time teachers [hr/week]	44.9 ± 8.7		45.1 ± 8.8		44.8 ± 8.4		44.5 ± 8.6		2.52	0.081	<0.01
Part-time teachers [hr/week]	36.5 ± 9.7		36.1 ± 10.6		35.8 ± 9.3		38.4 ± 8.9		28.83	<0.001	0.03

*n*, number of teachers. %: frequency in %. *χ*^2^-test by Pearson (test size: *χ*^2^-value, effect size: *d*). *M ± SD*: means ± standard deviations. General linear model (univariate, internal subject design: constant term + age group; test variable: *F*-value, df = 2, error def = 10,735, effect size: *η*^2^_p_). Significance (two-sided): *p* < 0.001, *p* > 0.05 (not significant). Effect size *d*: <0.20 = no effect, 0.20–0.49 = small effect, 0.50–0.79 = medium effect, ≥0.80 = large effect (57). Effect size *η*^2^_p_ (partial eta square): <0.01 = no effect, 0.01–0.06 = little effect, 0.06–0.14 = medium effect, ≥0.14 = large effect (57).

The sociodemographic data for the age groups were only comparable in terms of partnership status (*d* = 0.10). Most teachers lived in a stable partnership (85%). More than half of them (53%) still had children living in their own household. This was the case for almost three-quarters (72%) of those aged 41–50, but only 44% of those aged 51–60 (*d* = 0.32 - small effect). Six percent of teachers reported having to care for relatives in their household, with this proportion increasing significantly from AG_31-40_ (2%) to AG_51-60_ (14%) (*d* = 0.36 - small effect).

The weekly working hours showed a high degree of individual variation among both full-time and part-time teachers. While there was no statistically significant difference in teaching hours per week and total working hours per week among full-time teachers in the three age groups (*p* = 0.059/0.081), the part-time teachers in the AG_31-40_ worked about 2.5 hr/week less than their older colleagues in the AG_51-60_ (*η*^2^_p_ = 0.03 – small effect).

#### 2.4.1 Reference sample

The reference sample from the ‘study on mental health at work’ (51) consisted of 3,870 employed individuals (51% male) divided into the age groups (31–40 (24%), 41–50 (42%), 51–60 (34%) years). Almost three quarters (73%) of the sample worked at least 35 h/week (full-time), 24% worked part-time (14–34 hr/week) and 4% were marginally employed. Forty-one percent reported their highest school degree as a secondary school leaving certificate, while just under a third (32%) of those in employment had a university entrance qualification. Half of the sample (51%) reported having a vocational or technical certificate and 12% had a university degree (48).

## 3 Results

**Figure 1:**
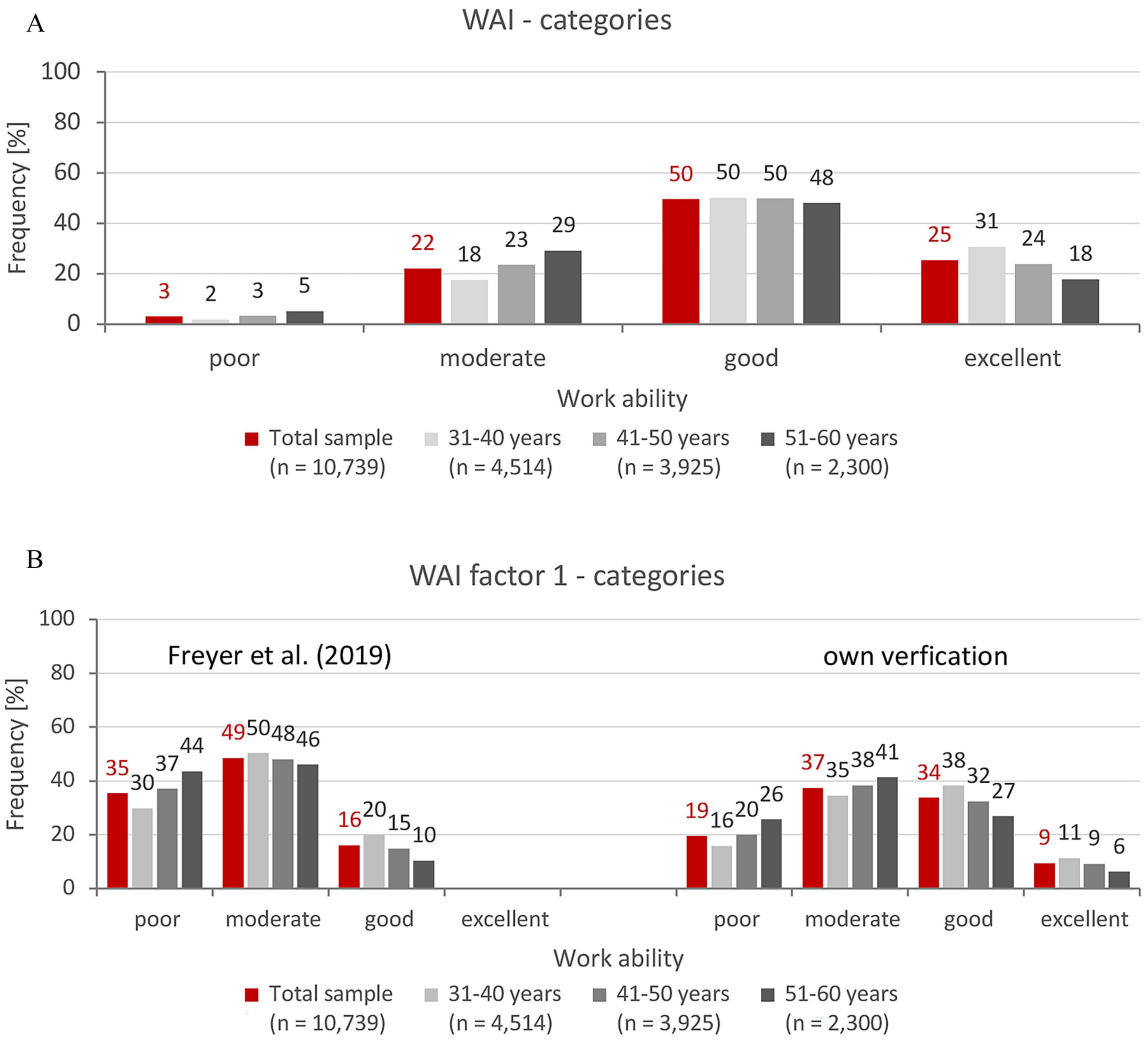
**(A)** Categories of Work Ability Index (WAI) of teachers (*n* = 10,739). *n*, number of teachers. Categorization of WAI according to Tuomi et al. (50) into poor (7–27 pts), moderate (28–36 pts), good (37–43 pts) and excellent (44–49 pts) work ability. **(B)** Categories of Work Ability Index (WAI) factor 1 of teachers (*n* = 10,739). Freyer et al. (47): categorization of WAI factor 1 based on a representative sample of employees according to Tuomi et al. (50) into poor (4–22 pts), moderate (23–26 pts), good (27–30 pts) and excellent (31 pts) work ability. Own verification: categorization of WAI factor 1 based on the current sample of teachers according to Tuomi et al. (50) into poor (4–20 pts), moderate (21–24 pts), good (25–27 pts) and excellent (28–31 pts) work ability.

First, the level of work ability among German teachers in three age groups is reported. To this end, the sum score WAI, its subscales and categories are considered and, analogously, the results for WAI factor 1 (WAI F1) are shown in Table 2 and Figures 1A,B. The work ability factor 1 of teachers is then compared with that of the German working population (Table 3).

**Table 2:** Main effects of work ability and its subscales of teachers.

			**Age group** [years]			**Significance**		
**Work ability**	**Dimension**	Total sample (*n* = 10,739)	31–40 (*n* = 4,514)	41–50 (*n* = 3,925)	51–60 (*n* = 2,300)	Test value	*p*-value	Effect size
Work Ability Index (WAI) [7–49 pts]	*M ± SD*	39.1 ± 5.6	40.3 ± 5.1	39.1 ± 5.5	37.9 ± 5.8	151.56	<0.001	0.03
Gender						62.35	<0.001	0.01
WAI factor 1 [4–31 pts]	M ± SD	23.3 ± 3.5	23.9 ± 3.3	23.2 ± 3.5	22.6 ± 3.6	107.82	<0.001	0.02
Gender						58.59	<0.001	0.01
WAI 1: current WA compared with the life time best [1 item with 0–10 pts]	*M ± SD*	7.7 ± 1.7	7.8 ± 1.7	7.6 ± 1.8	7.4 ± 1.8	56.89	<0.001	0.01
Gender						370.36	<0.001	<0.01
WAI 2: WA in relation to job demands [2 items with a sum of 2–10 pts]	*M ± SD*	7.5 ± 1.4	7.7 ± 1.4	7.5 ± 1.4	7.3 ± 1.4	46.98	<0.001	0.01
Gender						59.29	<0.001	0.01
WAI 2a: physical demands [1–5 pts]	*M ± SD*	3.9 ± 0.8	4.1 ± 0.8	3.9 ± 0.8	3.8 ± 0.8	129.88	<0.001	0.02
Gender						58.79	<0.001	0.01
WAI 2b: mental demands [1–5 pts]	*M ± SD*	3.7 ± 0.8	3.8 ± 0.8	3.7 ± 0.8	3.6 ± 0.8	19.49	<0.001	<0.01
Gender						43.89	<0.001	<0.01
WAI 6: estimation of own WA 2 years from now [1 item with 1, 4, 7 pts]	1: n (%) 4: n (%) 7: n (%)	18 (0.2) 403 (3.8) 10,318 (96.0)	10 (0.2) 95 (2.1) 4,409 (97.7)	4 (0.2) 132 (3.4) 3,789 (96.4)	4 (0.2) 176 (7.7) 2,120 (92.1)	134.19	<0.001	0.22
Gender						0.85	0.358	0.19
WAI 7: mental resources [3 items with a sum of 0–4 pts]	*M ± SD*	3.2 ± 0.8	3.2 ± 0.8	3.2 ± 0.8	3.2 ± 0.8	2.90	0.055	<0.01
Gender						<0.01	0.973	<0.01

WAI 6: 1 = unlikely, 4 = not certain, 7 = relatively certain. *n*, number of teachers. %: frequency in %. *χ*^2^-test by Pearson (test size: *χ*^2^-value, effect size: *d*). *M ± SD*: means ± standard deviations. General linear model (univariate, internal subject design: constant term + age group + gender, test variable: *F*-value, df = 2, error def = 10,735, effect size: *η*^2^_p_). Significance (two-sided): *p* < 0.001, *p* > 0.05 (not significant). Effect size *d*: <0.20 = no effect, 0.20–0.49 = small effect, 0.50–0.79 = medium effect, ≥0.80 = large effect (57). Effect size *η*^2^_p_ (partial eta square): <0.01 = no effect, 0.01–0.06 = small effect, 0.06–0.14 = medium effect, ≥0.14 = large effect (57).

**Table 3:** Comparison of work ability index factor 1 (WAI factor 1) with the reference sample.

Sample	Age group [years]	Teacher group			Reference group			Significance		
		WAI factor 1			WAI factor 1					
		*n*	*M*	*SD*	*n*	*M*	*SD*	Test value	*p*-value	Effect size *d*
Total sample	31–60	10,739	23.3	3.5	3,870	25.7	3.9	69.79	<0.001	0.67
Male	31–40	1,540	24.4	3.2	518	26.6	3.2	27.54	<0.001	0.70
	41–50	1,229	23.4	3.5	811	25.7	3.8	22.53	<0.001	0.64
	51–60	663	22.9	3.7	649	24.9	4.2	13.72	<0.001	0.53
Female	31–40	2,974	23.6	3.3	422	26.6	3.7	49.18	<0.001	0.90
	41–50	2,696	23.1	3.5	806	26.0	3.9	42.34	<0.001	0.82
	51–60	1,637	22.4	3.6	664	25.0	3.9	29.15	<0.001	0.72

WAI factor 1 [4–31 pts]. *n*, number of teachers and employees. *M*, means; *SD*, standard deviations. One sample *t*-test (test value: *t*-value, effect size: *d*). Significance (two-sided): *p* < 0.001. Effect size *d*: <0.20 = no effect, 0.20–0.49 = small effect, 0.50–0.79 = medium effect, ≥0.80 = large effect (57). Reference sample (*n* = 3,870 employees): sample of the ‘study on mental health at work’ (48, 51). The sample of the ‘study on mental health at work’ was drawn from the integrated employment biographies of the German Federal Employment Agency, which is held by the Institute for Employment Research. Data collection was performed by the Institute for Applied Social Sciences. Data access to the scientific use file of the ‘study on mental health at work’ can be applied for at the research data Center of the Institute for employment research (http://fdz.iab.de/de/FDZ_Individual_Data/SMGA.aspx).

The results of the univariate covariance analyses of work-related factors (subscales of ERI-Q) and personal factors (OC, EE) for the three age groups and the risk factors (*χ*^2^-test) are then presented.

**Figure 2:**
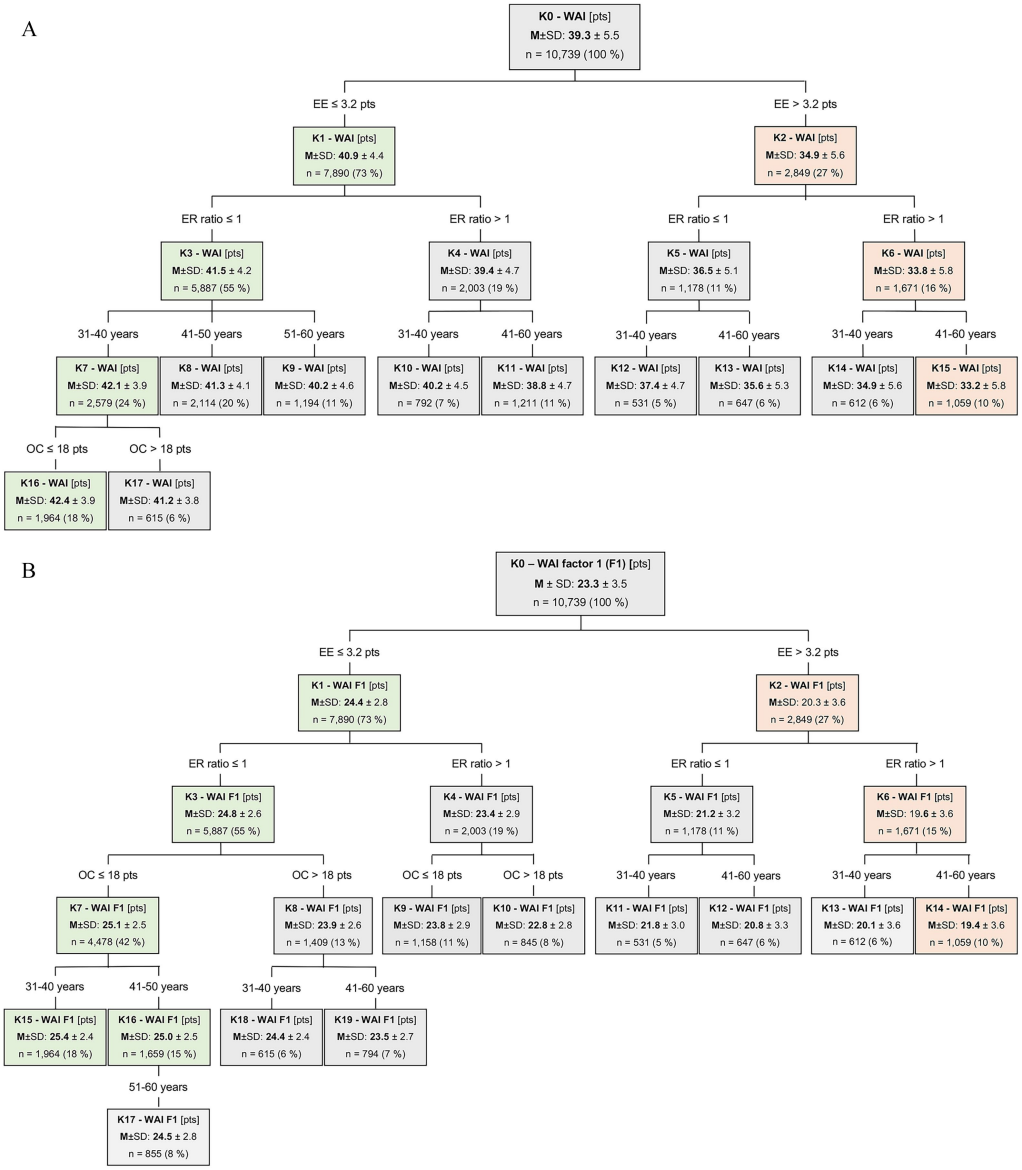
**(A)** Decision tree of Work Ability Index (WAI) of teachers (*n* = 10,739). K, decision node; pts, points; *n*, number of teachers; *M ± SD*, means ± standard deviations; ERI, effort-reward imbalance; ER ratio, effort-reward ratio; OC, overcommitment; EE, emotional exhaustion. **(B)** Decision tree of Work Ability Index factor 1 (WAI F1) of teachers (*n* = 10,739). K, decision node; pts, points; *n*, number of teachers; *M ± SD*, means ± standard deviations; ERI, effort-reward imbalance; ER ratio, effort-reward ratio; OC, overcommitment; EE, emotional exhaustion.

Finally, the results for determining the predictors of work ability are presented. This includes both the correlation and the regression analyses (Table 4). In addition, CHAID analyses (59) are used to visualize the complex relationships between the predictors and the target variables (WAI, WAI F1) in the form of decision trees (Figures 2A,B).

**Table 4:** Regression models of work-related and personal factors of the work ability of teachers (*n* = 10,739).

Model		Non-standardized coefficient		Standardized coefficient *β*	Significance		95% CI for *B*		Corrected *R*^2^
		Regression coefficient *B*	Standard error *B*		*t*-value	*p*-value	Lower limit	Upper limit	
	Work Ability Index (WAI)								
1	(Constant)	44.35	0.12		366.86	<0.001	44.12	44.59	
	ER ratio	−5.47	0.12	−0.40	−45.32	<0.001	−5.71	−5.23	0.16
2a	(Constant)	50.12	0.25		199.50	<0.001	49.63	50.62	
	OC score [pts]	−0.62	0.01	−0.39	−43.79	<0.001	−0.65	−0.59	0.15
2b	(Constant)	45.37	0.09		515.41	<0.001	45.20	45.55	
	EE score [pts]	−2.60	0.03	−0.60	−78.14	<0.001	−2.67	−2.54	0.36
3a	(Constant)	44.70	0.27		165.43	<0.001	44.17	45.23	
	Age [years]	−0.12	0.01	−0.19	−20.25	<0.001	−0.14	−0.11	0.04
3b	(Constant)	40.95	0.20		208.59	<0.001	40.56	41.33	
	Gender (male = 1, female = 2)	−0.96	0.11	−0.08	−8.55	<0.001	−1.18	−0.74	0.01
	Total model					<0.001			
4	(Constant)	52.69	0.29		181.29	<0.001	52.12	53.26	
	ER ratio	−1.91	0.12	−0.14	−16.58	<0.001	−2.14	−1.69	0.42
	OC score [pts]	−0.12	0.01	−0.07	−8.41	<0.001	−0.14	−0.09	
	EE score [pts]	−2.15	0.04	−0.50	−55.31	<0.001	−2.23	−2.08	
	Age [years]	−0.11	0.01	−0.16	−22.02	<0.001	−0.12	−0.10	
	WAI factor 1								
1	(Constant)	26.71	0.08		348.45	<0.001	26.56	26.86	
	ER ratio	−3.67	0.08	−0.42	−47.84	<0.001	−3.81	−3.51	0.18
2a	(Constant)	30.92	0.16		195.84	<0.001	30.61	31.22	
	OC score [pts]	−0.43	0.01	−0.43	−48.85	<0.001	−0.45	−0.42	0.18
2b	(Constant)	27.49	0.05		510.44	<0.001	27.39	27.60	
	EE score [pts]	−1.79	0.02	−0.65	−87.64	<0.001	−1.83	−1.75	0.42
3a	(Constant)	26.27	0.17		151.11	<0.001	25.92	26.60	
	Age [years]	−0.07	<0.01	−0.16	−17.09	<0.001	−0.08	−0.06	0.03
3b	(Constant)	24.34	0.13		193.76	<0.001	24.10	24.59	
	Gender (male = 1, female = 2)	−0.59	0.07	−0.08	−8.22	<0.001	−0.73	−0.45	0.01
	Total model								
4	(Constant)	31.96	0.18		178.93	<0.001	31.61	32.31	
	ER ratio	−1.20	0.07	−0.14	−16.92	<0.001	−1.34	−1.06	0.46
	OC score [pts]	−0.10	0.01	−0.10	−11.39	<0.001	−0.11	−0.08	
	EE score [pts]	−1.48	0.02	−0.53	−61.75	<0.001	−1.52	−1.43	
	Age [years]	−0.06	<0.01	−0.13	−18.74	<0.001	−0.06	−0.05	

*B*, beta-coefficient; CI, confidence interval; ER ratio, effort-reward ratio; OC, overcommitment; EE, emotional exhaustion. Multiple linear regression (method: enter), dependent variable: work ability (WAI, WAI F1). Significance (two-sided): *p* < 0.001.

Full-time and part-time teachers were included in the statistical analyses as a total sample, as the preliminary analysis did not reveal any practically significant group effects for work ability or work-related and personal factors between full-time and part-time teachers.

### 3.1 Work ability

The results for work ability (WAI, WAI F1) for the three age groups are summarized in Table 2 and in Figure 1A (WAI categories) and Figure 1B (WAI F1 categories).

There was a significant difference between the age groups in terms of work ability (WAI, WAI F1: *η*^2^_p_ = 0.03/0.02 – small effect): AG_31-40_ reported higher work ability than AG_51-60_ (WAI: *M* = 40 vs. 38 of 49 pts, WAI F1: *M* = 24 vs. 22 of 31 pts), with these WAI averages in the good range in all age groups.

This effect was observed for the WAI 1, WAI 2, and WAI 6 subscales of WAI factor 1, according to which AG_31-40_ showed significantly better current work ability (WAI 1: *η*^2^_p_ = 0.01 – small effect) and task management (WAI 2: *η*^2^_p_ = 0.01 – small effect) compared to AG_51-60_. Most teachers (96%) could imagine continuing in their current job over the next 2 years (WAI 6). As expected, an age group effect was observed here; significantly more younger teachers (AG_31-40_) than older teachers (AG_51-60_) confirmed this statement (*d* = 0.22 - small effect). The mental resources subscale (WAI 7) did not differ significantly between age groups (WAI 7: *η*^2^_p_ < 0.01).

Significant gender effects were found for both the WAI and the WAI factor 1 (*η*^2^_p_ = 0.01 - small effect). Accordingly, men (*n* = 3,432) had a significantly higher WAI than women (*n* = 7,307) (*M* = 40 vs. 39 of 49 pts, *SD* = 5.4 vs. 5.5 pts; *F*(1) = 73.12, *η*^2^_p_ = 0.01 – small effect). The same effect was found for WAI factor 1 (*M* = 24 vs. 23 of 31 pts, *SD* = 3.5 pts; *F*(1) = 58.56, *η*^2^_p_ = 0.01 – small effect). Unexpectedly, at the individual item level, a significant gender effect was found only for WAI 2 (*η*^2^_p_ = 0.01 – small effect), but only in coping with physical demands (*η*^2^_p_ = 0.01 – small effect); men coped better than women. No practically significant gender effects were found for the WAI 1, WAI 6, and WAI 7 subscales (*η*^2^_p_ < 0.01, *d* = 0.19).

The significant difference between age groups was also affirmed for the WAI categories [*χ*^2^(6) = 259.10, *p* < 0.001, *d* = 0.31 – small effect]. Around 80% of teachers in the AG_31-40_ reported good and excellent work ability (37–49 pts), but this was only the case for two-thirds (66%) of teachers in the AG_51-60_. Poor work ability was indicated by 2% of teachers in the AG_31-40_ and 5% in the AG_51-60_.

When teachers were categorized for WAI factor 1 based on data from the reference sample and Freyer’s recommendations (47), a high proportion of teachers with poor work ability was noticeable, rising from 30 to 44% with increasing age. The proportion of teachers with moderate work ability decreased slightly with increasing age, from 50 to 46%. Only 20% of teachers in the AG_31-40_ and 15 and 10% in the two higher age groups (AG_41-60_) fell into the category of good work ability, and none of the teachers had excellent work ability (Figure 1B, left). A significant age group effect was validated for WAI factor 1 [*χ*^2^(4) = 183.20, *p* > 0.001, *d* = 0.26 – small effect].

However, when the WAI factor 1 of teachers was categorized based on the data from the present study and the guidelines of Tuomi et al. (50) (Figure 1B, right), an age effect was also found [*χ*^2^(6) = 201.63, *p* < 0.001, *d* = 0.28 – small effect]. However, the overall picture was different: the proportion of teachers with excellent work ability decreased with age, but was still 11% (AG_31-40_) and 6% (AG_51-60_). The proportion was also significantly higher in the good work ability category in all age groups than in Freyer’s categorization (48), but as expected, decreased with age from 38% (AG_31-40_) to 27% (AG_51-60_). In contrast, the proportion of teachers with moderate and poor work ability increased with age from 35 to 41% and from 16 to 26%, respectively.

Comparing the two categorizations of WAI factor 1, it is noticeable that the data from the reference sample lead to a left-skewed distribution, while the values from this study’s sample are closer to a normal distribution.

### 3.2 Work ability compared to the reference sample

The results comparing the work ability (WAI, WAI F1) of teachers with that of the German working population (48) are summarized below (Table 3).

The WAI did not differ significantly between teachers and the reference sample (*M* = 39 vs. 40 of 49 pts, *SD* = 5.6 vs. 6.2 pts; *t* (10,738) = 16.51, *d* = 0.16), while the WAI factor 1 in the reference sample showed significantly higher work ability (*M* = 23 vs. 26 of 31 pts, *SD* = 3.5 vs. 3.9 pts; *t*(10,738) = 69.79, *d* = 0.67 – medium effect).

Regardless of gender, teachers in all age groups showed poorer work ability for WAI factor 1 than the German working population (*d* = 0.53–0.90 – medium–large effects). A gender effect was observed for WAI factor 1 among teachers only in AG_31-40_ [*F*(1) = 54.28, *p* < 0.001, *η*^2^_p_ = 0.01 – small effect], i.e., in AG_41-60_ there was no practically significant difference between male and female teachers for the mean values of WAI factor 1 [AG_41-50_: *F*(1) = 41.50, *p* = 0.008, *η*^2^_p_ < 0.01; AG_51-60_: *F*(1) = 8.80, *p* = 0.003, *η*^2^_p_ < 0.01].

### 3.3 Work-related and personal factors

To determine the psychosocial workload and the influence of personal factors on work ability (WAI, WAI F1), the subscales of the ERI-Q (23) were used, including the intrinsic factor overcommitment (OC) and the core component of burnout risk, emotional exhaustion (EE) (54). The results are summarized in (see both under Supplementary material).

The mean values of the ER subscales Effort and ER Ratio differed significantly between the age groups (*η*^2^_p_ = 0.02/0.01 – small effects). On average, the AG_31-40_ showed significantly lower effort than the two older age groups (AG_41-60_) (*M* = 9 vs. 10 of 15 pts), while the reward did not differ between the age groups (*M* = 26 of 35 pts), (*η*^2^_p_ < 0.01). This applied to professional advancement opportunities, appreciation or recognition, and job security (*η*^2^_p_ < 0.01).

The mean ER ratio for teachers was 0.92, which is still outside the risk range (23). This ER ratio was significantly lower in the AG_31-40_ (*M* = 0.89) than in the two older age groups (*M* = 0.94, *η*^2^_p_ = 0.01 – small effect). A health risk (ER ratio > 1) was present for more than one-third (34%) of teachers, but did not differ significantly between age groups in practical terms [*χ*^2^(2) = 35.16, *p* < 0.001, *d* = 0.12].

There were also no significant differences in the mean values for OC and EE between the age groups (*η*^2^_p_ < 0.01). At 18 pts, they were still classified as normal for OC (23), but were close to the threshold for the noticeable range (19–24 pts). A critical OC value (>18 pts) was observed in significantly more than one third (39%) of teachers [*χ*^2^(2) = 19.73, *p* < 0.001, *d* = 0.11], i.e., these teachers can be assumed to be overexerting themselves, which may be associated with an increased health risk.

For EE, the average score for teachers was 2.3 pts, which was also within the normal range (53). Elevated EE values (≥3.2–6.0 pts) were reported by about a quarter (27%) of teachers [*χ*^2^(2) = 34.84, *p* < 0.001, *d* = 0.09].

Neither work-related n`or personal factors were significantly influenced by gender (*η*^2^_p_ < 0.01, *d* < 0.20). In summary, marginal age effects must be taken into account when determining psychosocial workload, while age and gender effects can be disregarded for personal factors.

### 3.4 Correlation analysis

Correlation analyses were first calculated to identify predictors of work ability (WAI, WAI F1). First, the associations (*R*) between age, work ability (WAI, WAI F1), ER ratio, OC, and EE were examined. This was followed by an examination of the associations with gender (*η*).

It was found that there was only a very slight negative association between age and work ability (WAI, WAI F1) (*R* = −0.19/−0.16) and no association with work-related and personal factors (*R* = 0.07/-0.03). Gender correlated only very slightly with work ability (WAI, WAI F1: *η* = 0.12/0.11), ER ratio (*η* = 0.15) and OC (*η* = 0.15) and not at all with EE (*η* = 0.06).

There were weak negative associations between work ability (WAI, WAI F1) and ER ratio (*R* = -0.40/−0.42) and OC (*R* = −0.39/−0.43), while moderate associations were found with emotional EE (*R* = −0.60/−0.65). There were only weak associations between ER ratio and OC (*R* = 0.40) and EE (*R* = 0.44), and a moderate association between OC and EE (*R* = 0.51).

This suggests that the lower the effort-reward ratio, tendency to overcommitment, and emotional exhaustion, the higher the work ability.

### 3.5 Predictors of work ability

To clarify whether work-related (ER ratio) and personal factors (OC, EE) or control variables (gender, age) represent relevant predictors of work ability (WAI, WAI F1), each variable was first subjected to a simple linear regression. In a second step, a multiple linear regression was used to create the overall model (method: enter) containing the significant predictors. These results are summarized in Table 4.

All models examined were statistically significant, meaning that every work- and person-related predictor, including age and gender, had a significant influence on work ability – both on the WAI [*F*(1) = 73.12–6,101.36: *p* < 0.001] and the WAI factor 1 [*F*(1) = 67.68–7,675.34, *p* < 0.001].

Upon closer examination of each individual predictor (models 1-3b), EE was verified as the most important predictor of work ability, explaining 36% (WAI) and 42% (WAI factor 1) of the variance, while the ER ratio or OC explained only 15 to 18% of this variance. Gender and age proved to be of secondary importance in this context (corrected *R*^2^: WAI = 0.01–0.04, WAI F1 = 0.01–0.03).

Since gender in particular contributed little to explaining the variance in work ability (WAI, WAI F1), only the ER ratio, OC, EE and age were included in the overall models. For these four predictors (models 4), the significant (*p* < 0.001) influence on the predictive performance of the model and thus on work ability (WAI, WAI F1) was confirmed. A variance explanation of 42% (WAI) and 46% (WAI F1) was achieved, which is considered an acceptable fit between the model and the data (60).

If all other independent variables in the overall model were held constant, the WAI decreased by 1.9 pts with each increase in the ER ratio by one unit. When EE increased by one point, the WAI decreased by 2.2 pts. This effect is significantly lower for OC and age. Similar results were obtained for WAI factor 1: with each increase in the ER ratio by one unit or in EE by one point, WAI factor 1 and thus work ability decreased by 1.2 and 1.5 pts, respectively.

### 3.6 Decision trees for work ability

The decision tree shows how work ability changes when the predictors (categories of ER ratio, OC, EE, age groups) change. The total sample (top node) is first divided into categories of the best predictor. This algorithm runs in each branch of the resulting tree until no more significant predictors are found. The higher the level, the more significant the explanatory value of the predictors. The tree structure was influenced by the specification of a maximum tree depth of four and a minimum number of 500 cases per node (segment). The decision trees for WAI and WAI factor 1 are shown in Figures 2A,B.

In both decision trees, EE was identified as the predictor most strongly associated with work ability (WAI, WAI F1). When EE was high or normal, 35 or 41 pts were expected for the WAI and 20 or 24 pts for the WAI factor 1. The ER ratio proved to be the second strongest predictor in each case. OC was not relevant in association with high EE. Only in teachers with normal EE and unremarkable ER ratios was there an association between OC and work ability.

In the WAI decision tree, moderate work ability (28–36 pts, on the right) was associated with high EE, ER ratio, and the three age groups. The lowest WAI (*M* = 33 pts) was reported for high EE and an ERI in the AG_51-60_ (node 15), affecting 10% of teachers. High EE alone was associated with relatively low work ability (*M* = 35 pts) in 6% of teachers in the AG_41-60_ (node 13). The best work ability (*M* = 42 pts) was found in the AG_31-40_ (node 16) for 18% of teachers. Similarly high WAI values (*M* = 41 pts) were also observed in node 1 (normal EE), node 3 (normal EE + normal ER ratio) or node 8 (normal EE + normal ER ratio + AG_41-50_) and even in node 17 (normal EE + normal ER ratio + AG_31-40_ + high OC), whereby the structure of node 8 applied to 20% and that of node 17 to only 6% of teachers.

For the WAI factor 1, the worst mean value (*M* = 19 of 31 pts) was again found for 10% of teachers with a combination of high EE and ERI in AG_41-60_ (node 14). The best work ability (*M* = 25 of 31 pts) was reported by teachers with normal values for EE, ER ratio, and OC in the age range between 31 and 50 years (nodes 15 and 16), which affected about one third (34%) of the sample.

## 4 Discussion

This study examines a representative sample of German upper secondary school teachers with regard to their work ability and suitable predictors. For the first time, in addition to the established unweighted WAI, the WAI factor 1 was used, which is based on the assumption of a two-factor structure of the WAI (47).

Exhaustion is the most important predictor of work ability, with a variance explanation of 36% (WAI) and 42% (WAI F1), while psychosocial work stress and overcommitment explain only 15 to 18% of the variance. Although age and gender are also significant predictors of work ability, they are of minor importance.

Emotional exhaustion is also highlighted as the most important classification feature in the decision trees. The decision trees show the effects of each predictor and their combinations with subordinate, less relevant predictors on work ability. This could be of interest because precise point values for the WAI or WAI factor 1 for teachers have hardly been published to date. In the earlier study by Seibt et al. (5), some burnout symptoms for teachers also resulted in an average WAI of 39 points. If no burnout symptoms were present, the average WAI for teachers improved to 41 points, meaning that both values were in the range of good work ability.

The study shows that 10% of older teachers (AG_51-60_) who report high levels of exhaustion and an imbalance between work effort and rewards belong to the group with the lowest work ability. The result is consistent with previous studies on various occupational groups in which psychosocial work stress has been negatively associated with work ability independently of and beyond other variables (24, 31). In particular, the longitudinal study by Bethge et al. (24) supports these findings, as psychosocial stress also had a greater influence on work ability than age in this study. Seibt et al. (5) found the best work ability (WAI: *M* = 44 pts) among office workers without burnout symptoms and with a lower age. For secondary school teachers, age was of minor importance in this association. Overall, female teachers had a 1.6 times higher risk of reduced work ability than office workers.

The practical significance of a predictor is clearly illustrated by the example of overcommitment. This unfavorable work behavior is only relevant for work ability as a secondary predictor in cases where emotional exhaustion is normal. Previous studies have associated overcommitment with low work ability (31, 61, 62). In a Swedish longitudinal study of public health service employees, overcommitment in combination with imbalance in everyday life in women and low educational level in men predicted reduced work ability (32).

Based on the WAI, teachers show good work ability on average, which does not differ significantly from that of the reference sample of the German working population. However, if the WAI factor 1 is applied, the work ability of teachers of all age groups, regardless of gender, is significantly lower than that of the general working population. Professional demands may have a negative impact on teachers’ work ability (63–65), especially when the necessary resources to cope are lacking.

Otherwise, occupational check-ups show that teachers are a relatively healthy professional group who behave in a more health-conscious manner than the general population. With the exception of high blood pressure, they have lower cardiovascular risk factors (66). However, these potential health benefits are explicitly excluded from consideration when using WAI factor 1 and cannot compensate for any ‘disadvantages’ resulting from occupational stress. Although stress-related mental illnesses are more common among teachers than in the general population (39, 67), they have a small impact on the WAI total score, accounting for only 1 of 14 diagnoses.

In line with previous research (5, 34, 67–69), the results also show a significant decline in work ability with increasing age. This effect is visible for both the WAI and the WAI factor 1. It is noteworthy that only mental resources (WAI 7) remain at the same level across all three age groups. It can be assumed that teachers are better able to cope with emotional demands at work as they become older and that this gain in resources enables them to better buffer negative consequences on work ability. Both the correlation and regression analyses as well as the CHAID analyses in this study confirm previous research findings that age has a consistent but only slight negative association with work ability overall.

If work ability is categorized based on the WAI score, there is also a significant difference between the age groups: around 80% in the AG_31-40_ report excellent or good work ability, while only two thirds of teachers in the AG _51–60_ do so. However, if our sample is categorized by the point value for the WAI factor 1 according to Freyer’s recommendations (48) and based on the reference sample from the general working population, a different scenario arises: although a significant age effect is also evident here, no teachers fall into the category of excellent work ability, and only 20% of 31- to 40-year-olds and 15 and 10% of the upper age groups (AG_41-60_) achieve good work ability. This discrepancy in the assessment of work ability between WAI and WAI factor 1 needs to be examined in more detail and requires further research.

If WAI factor 1 in the sample is categorized based on the recommendations developed for the WAI by Tuomi et al. (50), 30% of the sample falls into the categories of good and moderate work ability, and another 15% into the categories of excellent and poor work ability. Even though this results in a significantly more positive assessment of teachers’ work ability, the transfer of the WAI categorization to WAI factor 1 requires further interprofessional verification and subgroup analyses.

Former studies have, however, provided conflicting findings on the influence of gender on work ability. Some studies have reported a tendency toward lower work ability among women (24, 70, 71), which is supported by the results of this study: male teachers rated their work ability slightly better than their female colleagues.

With regard to the extent of psychosocial workloads, our findings are consistent with previous studies suggesting that teachers perceive a greater imbalance between effort and reward than other occupational groups (5). In this study, about one third of teachers feel such an imbalance, which is comparable to the findings of Mußmann and Hardwig (72, 73). The resulting psychobiological stress reactions with potential health consequences have been documented in a large number of studies (25, 74, 75). In addition, 39% of all teachers tend to be overexerted at work. It is assumed that the effects of an imbalance between effort and reward on health are particularly harmful to people who tend to be overcommitted to their work (26). Since teachers often extend their working hours into the evening and weekend (76), it is even more difficult for them to detach from work (77, 78). In line with this, the current study found that more than a quarter of all teachers scored noticeably high on emotional exhaustion. It also shows that emotional exhaustion in teachers is linked with overcommitted work behavior. As overcommitment is understood as a motivational pattern in which employees increase their efforts beyond the normal level and possibly overestimate the resources available to them (23), associations with exhaustion are explicable. This finding is supported by studies in which overcommitment was found to be significantly associated with burnout (62, 79).

Research on teachers repeatedly emphasizes that, in addition to the promotion of personal resources, a supportive working atmosphere and good interpersonal relationships with colleagues and superiors are essential work resources that have a positive influence on work ability (16, 80, 81).

### 4.1 Strength of the study

The uniqueness of the study lies in the fact that a very large and representative sample of German secondary school teachers was examined in terms of work ability, so that the data collected can be used as a reference. In addition, the less well-known WAI factor 1 was recorded alongside the established WAI, and the necessary further development of the categorization was worked out.

### 4.2 Limitations of the study

The cross-sectional design does not allow for an evaluation of causal associations between the variables. With regard to data collection, the results are subject to the known limitations of self-assessments (including biases due to social desirability, response tendencies, and memory deficits). Furthermore, data collection and instruction for the WAI are not standardized, which limits the objectivity of the study. In the present study, participants completed the questionnaire online, while in the reference sample, it was collected via telephone interview. The evaluation guidelines provided by the developers (50) were used to categorize both the WAI and the WAI factor 1. However, based on the reference sample, these guidelines lead to implausible results for the WAI factor 1 and need to be adjusted. The effort scale has only a low internal consistency of 0.52 (53).

Since participation in the study was voluntary, selection effects or the healthy worker effect cannot be excluded. This may have led to an underestimation of the effects. In addition, the generalizability of the WAI results above the age of 60 is limited, as both the sample of teachers and the reference sample from the general working population only contained data sets from employees up to the age of 60.

## 5 Conclusion

The study makes an essential contribution to understanding teachers’ work ability by highlighting not only working conditions but also the importance of individual work behavior and mental health in maintaining one’s own work ability. Emotional exhaustion emerged as the most important predictor of work ability and should be given special attention in occupational health check-ups.

Teachers need an adequate level of work ability to cope with the broad and ever-changing range of work tasks throughout their professional lives. Measures to promote teachers’ work ability should therefore take into account individual risks such as emotional exhaustion and overcommitment.

School administrators should take care to prevent overcommitment, work overload, and the tendency to extend working hours at the expense of adequate rest periods. In addition, they should promote the improvement of individual skills and opportunities for coping with stressful situations, improve social support, and strengthen teamwork.

From a methodological perspective, the categorization of WAI factor 1 needs to be further developed through subgroup analyses.

## Data Availability

The datasets presented in this article are not readily available because no transfer of data was agreed upon during data collection. Requests to access the datasets should be directed to steffi.kreuzfeld@uni-rostock.de.

## Supplementary material

SUPPLEMENTARY FIGURE 2 Risk assessment of work-related and personal factors of teachers (n = 10,739) Notes: n: number of teachers. ERI: effort-reward imbalance. ER ratio: effort-reward ratio. OC: overcommitment. EE: emotional exhaustion.
