# Supplementary figures and images for: Predictors of work ability of secondary school teachers in Germany
Source: Front Public Health. 2026 Feb 11;13:1708490. doi: 10.3389/fpubh.2025.1708490 (PMC12933950; doi:10.3389/fpubh.2025.1708490)

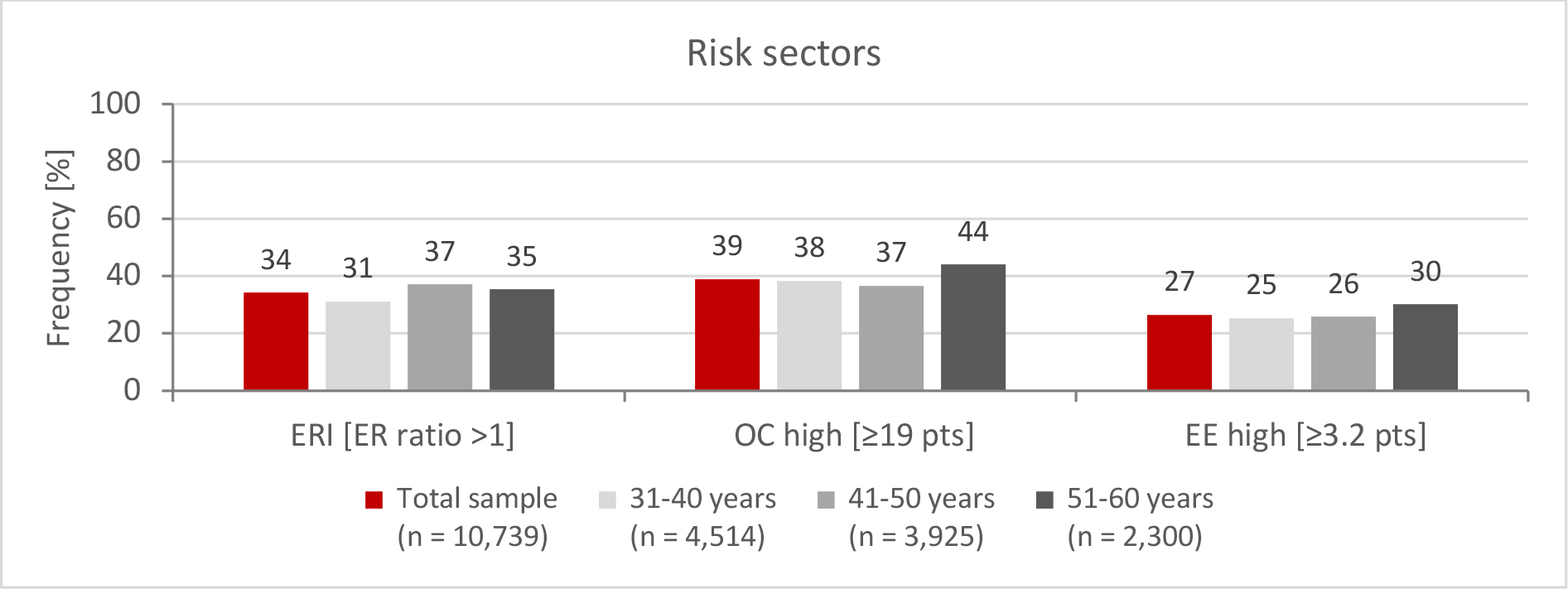

Supplement: Supplementary file 1 [file Image_2.jpg]
