# Supplementary material for: Predictors of work ability of secondary school teachers in Germany
Source: Front Public Health. 2026 Feb 11;13:1708490. doi: 10.3389/fpubh.2025.1708490 (PMC12933950; doi:10.3389/fpubh.2025.1708490)
Supplement: Supplementary file 2 [file Table_4.docx]

TABLE 4 | Main effects of work-related and personal factors and the covariate (gender) of teachers

|  |  | **Age group** [years] | | | **Significance** | | | |
| --- | --- | --- | --- | --- | --- | --- | --- | --- |
| **Subscale (factor)** | Total sample (n = 10,739) | 31-40  (n = 4.514) | 41-50  (n = 3.925) | 51-60  (n = 2.300) | Test value | p-value | Effect size |  |
|  | M ± SD | M ± SD | M ± SD | M ± SD |  |  |  |  |
| **Effort** [3-15 pts] | **9.6** ± 2.6 | **9.3** ± 2.6 | **10.0** ± 2.6 | **9.8** ± 2.5 | 83.81 | <0.001 | 0.02 |  |
| - Gender |  |  |  |  | 0.01 | 0.985 | <0.01 |  |
| **Reward** [7-35 pts] | **26.2** ± 5.3 | **26.3** ± 5.4 | **26.3** ± 5.2 | **26.0** ± 5.4 | 1.51 | 0.220 | <0.01 |  |
| - Gender |  |  |  |  | 0.28 | 0.597 | <0.01 |  |
| **Job promotion** [3-15] | **11.0** ± 3.0 | **10.9** ± 3.0 | **11.1** ± 2.9 | **11.0** ± 3.0 | 6.87 | 0.001 | <0.01 |  |
| - Gender |  |  |  |  | 18.69 | <0.001 | <0.01 |  |
| **Esteem** [2-10 pts] | **7.1** ± 2.3 | **7.1** ± 2.3 | **7.0** ± 2.3 | **7.1** ± 2.3 | 0.91 | 0.404 | <0.01 |  |
| - Gender |  |  |  |  | 25.73 | <0.001 | <0.01 |  |
| **Job security** [2-10 pts] | **8.2** ± 1.6 | **8.3** ± 1.7 | **8.1** ± 1.5 | **8.0** ± 1.6 | 23.11 | <0.001 | <0.01 |  |
| - Gender |  |  |  |  | 0.96 | 0.328 | <0.01 |  |
| **Effort-reward ratio** | **0.92** ± 0.40 | **0.89** ± 0.41 | **0.94** ± 0.39 | **0.94** ± 0.39 | 24.49 | <0.001 | 0.01 |  |
| - Gender |  |  |  |  | 0.95 | 0.331 | <0.01 |  |
| **Overcommitment** [6-24 pts] | **17.5** ± 3.4 | **17.4** ± 3.4 | **17.4** ± 3.4 | **17.8** ± 3.4 | 14.18 | <0.001 | <0.01 |  |
| - Gender |  |  |  |  | 223.26 | <0.001 | 0.02 |  |
| **Emotional exhaustion** [0-6 pts] | **2.3** ± 1.3 | **2.3** ± 1.2 | **2.3** ± 1.3 | **2.4** ± 1.3 | 7.68 | <0.001 | <0.01 |  |
| - Gender |  |  |  |  | 28.16 | <0.001 | <0.01 |  |

Notes: M ± SD: means ± standard deviations. General linear model (univariate, internal subject design: constant term + age group + gender, test variable: F-value, df = 2, error def = 10,735, effect size: η²_p_). Significance (two-sided): p ≤ 0.001, p > 0.05 (not significant). Effect size η²_p_ (partial eta square): <0.01 = no effect, 0.01-0.06 = small effect, 0.06-0.14 = medium effect, ≥0.14 = large effect (57).
